# Structure-directing effects of ionic liquids in the ionothermal synthesis of metal–organic frameworks

**DOI:** 10.1107/S2052252517008326

**Published:** 2017-06-30

**Authors:** Thomas P. Vaid, Steven P. Kelley, Robin D. Rogers

**Affiliations:** a Department of Chemistry, McGill University, 801 Sherbrooke St. West, Montreal, Quebec H3A 0B8, Canada; b 525 Solutions, Inc., 720 2nd Street, Tuscaloosa, Alabama 35401, USA

**Keywords:** metal–organic framework, MOF, ionic liquid, ionothermal synthesis, template

## Abstract

Traditional synthesis of metal–organic frameworks (MOFs) involves the reaction of a metal-containing precursor with an organic linker in an organic solvent at an elevated temperature, in what is termed a ‘solvothermal’ reaction. More recently, many examples have been reported of MOF synthesis in ionic liquids (ILs), rather than an organic solvent, in ‘ionothermal’ reactions. The high concentration of both cations and anions in an ionic liquid allows for the formation of new MOF structures in which the IL cation or anion or both are incorporated into the MOF. Most commonly, the IL cation is included in the open cavities of the MOF, countering the anionic charge of the MOF framework itself and acting as a template around which the MOF structure forms. Ionic liquids can also serve other structure-directing roles, for example, when an IL containing a single enantiomer of a chiral anion leads to a homochiral MOF, even though the IL anion is not itself incorporated into the MOF. A comprehensive review of ionothermal syntheses of MOFs, and the structure-directing effects of the ILs, is given.

## Abstract 2

The ‘ionothermal’ synthesis of metal–organic frameworks (MOFs) in ionic liquids (ILs) has led to a large number of new MOFs, often by incorporation of the IL cation as a templating counter-ion for an anionic MOF framework.

## 1. Introduction   

**Figure 1:**
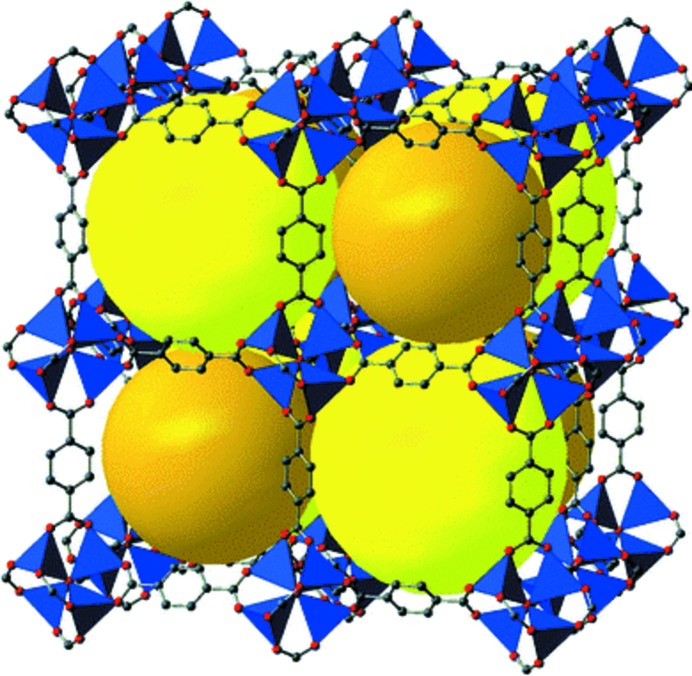
MOF-5, or Zn_4_O(BDC)_3_, with the yellow and orange spheres representing open space. From Hermes *et al.* (2006 ▸). Published by The Royal Society of Chemistry. Used with permission.

Metal–organic frameworks (MOFs) are crystalline framework materials that consist of metal atoms or cluster ‘nodes’ joined by multifunctional organic linking ‘struts’. The prototypical example, Yaghi’s MOF-5 (Li *et al.*, 1999 ▸) (see Fig. 1 ▸), serves to illustrate the important features of MOFs in general. MOF-5 has the formula Zn_4_O(BDC)_3_ (BDC^2−^ = benzene-1,4-dicarboxylate, the dianion of terephthalic acid) and contains two ‘secondary building units’ (SBUs), *i.e.* an oxygen-centered Zn_4_O cluster and the terephthalate dianion linker. The connection geometry of the SBUs that comprise a MOF often lead to a predictable structure for the MOF itself (Kim *et al.*, 2001 ▸), and that is the case for MOF-5. The Zn_4_O cluster connects to six carboxyl­ate groups in an octahedral arrangement, while the BDC^2−^ dianion connects to two Zn_4_O clusters in a linear arrangement. The combination of the two SBUs leads to the cubic open-framework structure of MOF-5. There are large open pores in MOF-5: the yellow and orange spheres of Fig. 1 ▸ represent these open spaces, with diameters of 15.1 and 11.0 Å, respectively, and the aperture between pores is large enough to allow passage of a sphere of diameter 8.0 Å (Li *et al.*, 1999 ▸). This accessible pore structure endows MOFs with their most interesting and potentially useful properties, and in fact MOFs are often *defined* to include only porous materials. MOFs have been examined as materials for hydrogen storage (Suh *et al.*, 2012 ▸), carbon dioxide capture (Sumida *et al.*, 2012 ▸; Liu *et al.*, 2012 ▸), gas separations (Banerjee *et al.*, 2015 ▸), chemical sensing (Kreno *et al.*, 2012 ▸), biomedical applications (Horcajada *et al.*, 2012 ▸), and many other applications (Zhou *et al.*, 2012 ▸).

There is some disagreement about what materials should be defined as MOFs. Omar Yaghi, who actually coined the term ‘metal–organic framework’, has suggested that it should refer to networks in which the bonds between metals and the organic linkers are formed with a functionality on the organic linker that would have a negative charge when not bound to the metal, as in a carboxylate, rather than be neutral, as in the N atoms of the pyridyl groups in 4,4′-bipyridine (Tranchemontagne *et al.*, 2009 ▸). Other functionalities that can form such bonds include imidazolate (and other azolates) and phosphonates. Such bonds are usually significantly stronger than the coordinate covalent or dative bonds formed by a pyridyl or similar moiety, which means that metal–carboxylate or similar bonds generally lead to more stable frameworks, but at the same time makes obtaining crystalline materials more challenging (Jiang *et al.*, 2016 ▸). Yaghi has suggested that the term *coordination polymer* be used to describe network materials formed by the weaker dative metal–ligand bonds, as in, for example, the diamondoid framework formed by the reaction of Cu^+^ and tetra(4-cyanophenyl)methane (Hoskins & Robson, 1989 ▸). On the other hand, an International Union of Pure and Applied Chemistry (IUPAC) recommendation on nomenclature suggested that a *coordination polymer* refer to any coordination compound with extended bonding in one, two, or three dimensions (with bonds of either type referred to above), a *coordination network* refer to a coordination polymer with extended bonding in two or three dimensions, and a *metal–organic framework* refer to a porous coordination network (Batten *et al.*, 2013 ▸). In any case, the vast majority of materials in this review are formed *via* metal–carboxylate linkages, and thus have the type of bonding appropriate for MOFs under Yaghi’s definition. Many are porous (or potentially porous) and are therefore MOFs under the IUPAC definition; however, many are not porous. Nevertheless, the nonporous inorganic–organic framework materials (usually based on carboxyl­ate linkages) are called MOFs by their creators, and are included in this review.

**Figure 2:**
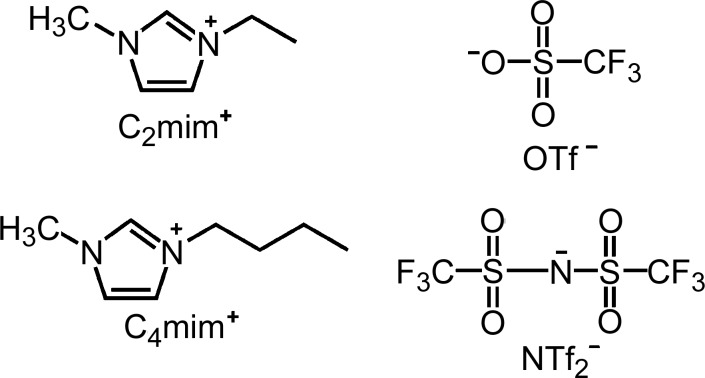
Structures of two common 1,3-dialkylimidazolium cations, *i.e.* [C_2_mim]^+^ and [C_4_mim]^+^, and two common anions, *i.e.* OTf^−^ and NTf_2_^−^, in ionic liquids.

The topic of this review is the use of ionic liquids (ILs) in the synthesis of MOFs. Ionic liquids are usually defined as materials that are composed entirely of ions and that have a melting point below 373 K (Rogers & Seddon, 2003 ▸). Some of the most common ILs are based on 1,3-dialkylimidazolium cations and weakly coordinating inorganic anions, such as tetrafluoroborate (BF_4_^−^), hexafluorophosphate (PF_6_^−^), tri­fluoro­methanesulfonate (triflate, OTf^−^) and bis­(tri­fluoro­methanesulfonyl)­imide (NTf_2_^−^) (see Fig. 2 ▸). Many of the 1,3-dialkylimidazolium cations are derived from 1-methylimidazole and thus have a methyl group as one of the alkyl substituents; a common abbreviation for such cations uses a C_*x*_ designation for the other alkyl substituent (*x* = the alkyl-chain length) and ‘mim’ for the methylimidazole portion, such that 1-ethyl-3-methylimidazolium is [C_2_mim]^+^ and 1-butyl-3-methylimidazolium is [C_4_mim]^+^ (see Fig. 2 ▸). While weakly coordinating and relatively chemically inert anions such as those listed above are most commonly used in ILs, several of the ILs in this review contain halide anions, such as chloride or bromide, which can result in the anions themselves being incorporated into the MOF framework.

A number of the properties of ILs are quite different from those of traditional solvents, and they can offer practical advantages and also access to new materials in the synthesis of MOFs. For example, ILs have extremely small or zero vapor pressure, which means that moderate-to-high-temperature synthetic reactions can be carried out in ILs with no need for either reflux condensers or a sealed system that can contain the autogenous pressure that would be generated with a traditional solvent. In contrast, a MOF synthesis in di­methyl­formamide (DMF), for example, would be limited in temperature range to the boiling point of DMF (425 K) with a reflux condenser, or would require a sealed vessel that can withstand the pressure generated by DMF at temperatures above its boiling point. The traditional synthesis of MOFs occurs in organic solvents at elevated temperatures, termed ‘solvothermal’ synthesis; by analogy, synthesis in ionic liquids is termed ‘ionothermal’ synthesis.

The most important property of ILs in the synthesis of MOFs is the presence of high concentrations of ions, and the opportunity to tune the properties of those ions to obtain new materials. Because charged ions are present, it is possible to create MOF frameworks with a net charge, with that charge counterbalanced by ions of the IL incorporated into the structure. For example, the 1,3-dialkylimidazolium cation of an IL can be incorporated into the pores of an anionic MOF framework. The anions of an IL can also become part of a MOF, usually coordinated to the metal center(s) of the MOF. In both cases, the ions have an important structure-directing effect. It is also possible for an IL to act simply as a solvent for MOF synthesis, with no part of the IL incorporated into the final structure. All of these situations will be discussed.

There were reviews published in 2007 and 2009 concerning the ionothermal synthesis of both MOFs and zeolites (Parnham & Morris, 2007 ▸; Morris, 2009 ▸), a review covering the use of ionic liquids in the synthesis of a variety of inorganic materials (including MOFs) (Freudenmann *et al.*, 2011 ▸), a review on the synthesis of MOFs in ILs, supercritical CO_2_, and IL/supercritical CO_2_ mixtures (Zhang *et al.*, 2016*a* ▸), and a review of the crystal engineering of MOFs, including a section on synthesis in ILs (Seoane *et al.*, 2016 ▸). However, none of these reviews were comprehensive on the topic of the ionothermal synthesis of MOFs, and this review will serve to update and expand upon the previous reviews.

This review is roughly organized by the type of structure-directing effect the IL has upon the MOFs, generally based on the part of the IL (cation, anion, neither, or both) that is incorporated into the MOF structure. However, some MOFs are discussed in a section to which they do not technically belong according to this categorization, such that they can be discussed in conjunction with related MOFs. Table 1 ▸ lists all the MOFs included in this review, along with the ILs in which they were synthesized, categorized by the section of the review in which they are discussed.

## 2. Ionothermal synthesis in which neither the cation nor anion of the IL is incorporated into the MOF   

As mentioned in the *Introduction* (§1), ILs have properties that can be advantageous over traditional solvents, such as their extremely low vapor pressures. They can thus be a useful replacement for organic solvents such as DMF, even when there is not a clear structure-directing role for the IL. Those syntheses will be explored in this section.

In our first example, the MOF Zn_4_(BTC)_2_(μ_4_-O)(H_2_O)_2_ was synthesized by the reaction of Zn(NO_3_)_2_·6H_2_O with benzene-1,3,5-tricarboxylic acid (H_3_BTC) in [C_2_mim][Br] (Xu *et al.*, 2008*c* ▸). It has a three-dimensional structure in which the [Zn_4_(μ_4_-O)(H_2_O)_2_]^6+^ SBU connects to six carboxylate groups, but not in the highly symmetric octahedral geometry observed in MOF-5 and other MOFs. Instead, two of the Zn^2+^ ions have coordinated water ligands and the asymmetry of the SBU leads to Zn_4_(BTC)_2_(μ_4_-O)(H_2_O)_2_ crystallizing in a non­centro­symmetric space group. It forms overall a (6,3) network in three dimensions. Neither the [C_2_mim]^+^ cation nor the Br^−^ anion is incorporated into the structure, but the choice of cation is important, as will be seen in later sections where other Zn–BTC MOFs do incorporate other imidazolium cations and adopt very different structures.

A set of MOFs containing polyoxometalate anions and other metal cations were synthesized in [C_2_mim][Br] (Fu *et al.*, 2012 ▸, 2011 ▸). One example is [NBu_4_]_2_[Cu(BBTZ)_2_(Mo_8_O_26_)] [BBTZ = 1,4-bis(1,2,4-triazol-1-ylmethyl)benzene]. Here, the [C_2_mim]^+^ cation was not incorporated into the MOF, but [NBu_4_]^+^ was, as [NBu_4_][Br] was added to the reaction mixture in anticipation of the need of the larger [NBu_4_]^+^ to fill the galleries of the MOF.

Two MOFs have been reported that incorporate the neutral molecule 1-methylimidazole (1-mim) into their structure, in both cases coordinated to a metal center and therefore clearly with a structure-directing effect. In one case, 1-mim was purposefully added as a reactant in the synthesis, where the reaction of Ni(NO_3_)_2_·6H_2_O, H_2_BDC, 1-mim, and NEt_3_ in [C_3_mim][Br] at 443 K yielded yellow–green (1-mim)Ni(BDC) (Hogben *et al.*, 2006 ▸). The nickel-containing nodes consist of two Ni(1-mim) units bridged by four carboxylate groups, forming a 4-connected square SBU, which combine with the linear BDC linkers to form a square net. The IL was critical to the formation of the MOF in significant yield and good purity — molecular solvents were unsuccessful. In a different synthesis, the three-dimensional (3D) MOF Zn_3_(BDC)_3_(1-mim)_2_ was synthesized in [C_4_mim][BF_4_], where the source of 1-mim is apparently decomposition of the [C_4_mim]^+^ cation (Wang *et al.*, 2008 ▸).

Reports occur fairly often in which the use of ILs in synthesis are emphasized, but the actual role of the IL is the straightforward replacement of another reagent in an otherwise standard solvent-based synthesis (Kelley & Rogers, 2015 ▸). In MOF synthesis, this can occur when an IL is dissolved in a conventional auxiliary solvent, making its role similar to any other linker ligand. Nevertheless, there are cases when the IL form of a reagent may confer advantages. For example, the solvothermal reaction of the aprotic IL [NEt_4_][2-methyl-4,5-dicyanoimidazolate] with Co(NO_3_)_2_·6H_2_O in DMF was used to form the zeolitic imidazolate framework (ZIF) Co[4,5-di(*N*-acetyl)amino-2-methylimdiazolate]_2_ (Mondal *et al.*, 2014 ▸). The reaction is essentially an *in situ* ligand synthesis, and neither IL ion is incorporated in the final structure. However, the use of neutral 4,5-dicyano-2-methylimidazole in this reaction failed to generate the ZIF, indicating that the role of the IL was to supply and stabilize the azole in its more reactive deprotonated form. Changing the counter-ion from [NEt_4_]^+^ to [C_8_mim]^+^ resulted in the crystallization of a more porous variant of the same structure with better gas-sorption properties, indicating that the cation is able to provide tunability despite not being involved in the reaction or directly incorporated in the structure (Mondal *et al.*, 2016 ▸).

A set of four zinc–imidazolate MOFs/ZIFs were synthesized ionothermally in [C_2_mim][NTf_2_] (Martins *et al.*, 2010 ▸). In no case was either the cation or the anion of the IL included in the MOF or ZIF, but varying the reaction conditions and zinc-containing precursors resulted in different products. Two of the compounds, *i.e.* Zn(C_2_O_4_)(C_3_N_2_H_4_) (C_2_O_4_ = oxalate and C_3_N_2_H_4_ = imidazole) and Zn(C_3_N_2_H_3_)_2_, were known pre­viously, but synthesized ionothermally for the first time. The MOF Zn(OAc)(C_3_N_2_H_3_) resulted from the reaction of Zn(OAc)_2_·2H_2_O and imidazole in [C_2_mim][NTf_2_] at 423 K for 48 h. Zn(OAc)(C_3_N_2_H_3_) has a 3D framework resulting from the bridging of both imidazolate and acetate linkers between tetrahedral Zn ions. The final compound is Zn_4_(C_3_N_2_H_3_)_8_(C_3_N_2_H_4_), formed from the reaction of Zn(NO_3_)_2_·6H_2_O and imidazole in [C_2_mim][NTf_2_] at 403 K for 48 h. The structure of Zn_4_(C_3_N_2_H_3_)_8_(C_3_N_2_H_4_) is a fairly complex 3D framework, but is notable mainly for the presence of terminal imidazole ligands. Here it is the reaction temperature that likely controls the structure formation, as reaction at 423 K leads to the fully condensed structure Zn(C_3_N_2_H_3_)_2_.

In another series of reactions, nanoparticles of Prussian blue, Fe_4_[Fe(CN)_6_]_3_, and a number of Prussian blue analogs, *M*_3_[Fe(CN)_6_]_2_ (*M* = Ni, Cu, Co), were synthesized in imidazolium ILs [C_*n*_mim][BF_4_] and [C_*n*_mim][Cl] (*n* = 2, 4, 10) (Clavel *et al.*, 2006 ▸; Larionova *et al.*, 2008 ▸). The synthesis of the Prussian blue analogs was performed through the combination of [*M*(H_2_O)_6_]^2+^ with [C_4_mim]_3_[Fe(CN)_6_] in [C_4_mim][BF_4_]. The water molecules of [*M*(H_2_O)_6_]^2+^ are displaced by the N atoms of [Fe(CN)_6_]^3−^ to form a 3D cubic network, as dictated by the octahedral coordination geometry of each metal center and the linear linkage of the cyanide ligands. Similar Prussian blue analogs have long been known (Entley & Girolami, 1994 ▸, 1995 ▸), but this synthesis in ILs leads to stabilized nanoparticles of the materials, likely through some type of electrostatic interaction of the IL ion(s) with the exterior of the particles.

## 3. IL cation incorporation/templating   

### 3.1. Introduction   

Long before the first MOFs were synthesized, zeolites were well known and industrially significant framework materials with porosity on the molecular scale (Sels & Kustov, 2016 ▸). Some zeolites are naturally occurring aluminosilicate minerals and many others have been synthesized in the laboratory and on an industrial scale. Within the aluminosilicate framework of a zeolite, both Si and Al atoms are bonded to four O atoms in a tetrahedral geometry, which places a net negative charge on the framework for each Al atom present. The negative charge of the framework is countered by weakly bound cations, often alkali cations such as Na^+^ or K^+^, that reside within the galleries, such that a typical zeolite formula would be Na_*x*_[(AlO_2_)_*x*_(SiO_2_)_*y*_]·*z*H_2_O. The alkali metal cations are often exchangeable with other cations, which leads to one of the important applications of zeolites, namely ion exchange. Zeolites are usually synthesized hydro­thermally from reactants such as sodium silicate and sodium aluminate in varying proportions. If a zeolite is synthesized with a tetra­alkyl­ammonium cation in place of the alkali-metal cation, the organic cation can act as a structural template, as it is incorporated into the zeolite galleries (Lok *et al.*, 1983 ▸), and it is possible to computationally design appropriate tetraalkylammonium cations for the synthesis of specific zeolites (Davis *et al.*, 2016 ▸). More recently, ILs have been used as the solvent in the ionothermal synthesis of zeolites, with the IL cations acting as structure-directing agents (Cooper *et al.*, 2004 ▸).

In an analogous manner, the ionothermal synthesis of MOFs in ILs allows the creation of anionic MOF frameworks with the cation of the IL incorporated into the galleries of the MOF to balance the charge and act as a template around which the framework forms. This is the most common way in which an IL acts as a structure-directing agent in MOF synthesis, and several examples will be described in this section.

### 3.2. Benzene-1,3,5-tricarboxylate (BTC^3−^) MOFs   

The reaction of Cd(NO_3_)_2_·4H_2_O with H_3_BTC in [C_2_mim][Br] at 433 K for 5 d yielded colorless crystals of a MOF with the simple stoichiometry of [C_2_mim][Cd(BTC)] (Liao *et al.*, 2006 ▸). The anionic [Cd(BTC)]^−^ framework contains Cd_2_ units with six carboxylate groups coordinated to them, four of which coordinate only one Cd cation and two of which bridge Cd cations. The overall connectivity can be viewed as an *MX*_2_-type, with *M* representing a Cd_2_ unit and *X* as the BTC^3−^ ligands. The authors of this study attempted ion exchange of the [C_2_mim]^+^ cations with Cu^2+^ and other metal cations, but those attempts were unsuccessful.

**Figure 3:**
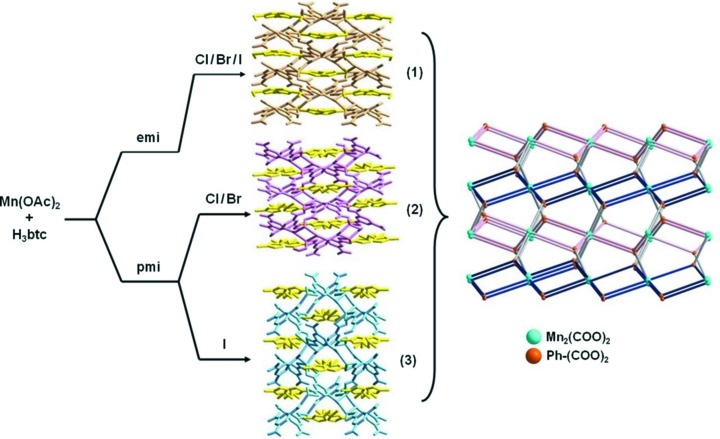
Structures of [C_2_mim][Mn(BTC)] (**1**) and [C_3_mim][Mn(BTC)] (**2** and **3**) crystallized from various [C_*n*_mim][*X*] (*n* = 2, 3; *X* = Cl, Br, I). From Xu *et al.* (2013 ▸). Published by the American Chemical Society. Used with permission.

A set of Mn–BTC MOFs demonstrate the interesting effects of the IL cation on the structure obtained, and also an influence of the anion even though the anion is not incorporated into the MOF structure (Xu *et al.*, 2013 ▸). The reaction of Mn(OAc)_2_·4H_2_O (OAc = acetate) with H_3_BTC in [C_2_mim][*X*] or [C_3_mim][*X*] (*X* = Cl, Br, I) in every case yields a compound with the formula [C_*n*_mim][Mn(BTC)] (*n* = 2, 3). However, there are three different structure types obtained (see Fig. 3 ▸), with [C_2_mim][*X*] yielding structure **1** for all *X* ^−^ anions, while [C_3_mim][*X*] gives structure **2** for *X* = Cl^−^ or Br^−^ and structure **3** for *X* = I^−^. The structures are somewhat complex when described in detail, but all contain Mn_2_ units and comprise an overall (3,6)-connected **pyr** topological network [see the *Reticular Chemistry Structure Resource* (O’Keeffe *et al.*, 2008 ▸) for topological notations]. Structure **1** is very similar to that of [C_2_mim][Cd(BTC)] mentioned above, with slightly different bonding in the *M*_2_ dimer but the same framework connectivity. The driving force for the formation of structure **1** *versus* **2** or **3** is clear, as the cavities within the anionic Mn(BTC)^−^ framework of **1** are smaller than those of **2** or **3**, such that **1** comfortably accommodates [C_2_mim]^+^ and **2** or **3** accommodate [C_3_mim]^+^ without extensive open space. The differentiation between structures **2** and **3** is more interesting and more difficult to rationalize. They have the same composition, [C_3_mim][Mn(BTC)], with no halide present, and somewhat different framework structures, and either **2** or **3** must be the thermodynamically more stable structure. However, with no halide present in the product, it must be a kinetic effect that leads to the formation of **2** for *X* = Cl^−^ or Br^−^ and **3** for *X* = I^−^. The syntheses were performed under identical conditions (except for the identity of the halide in the IL), so it is the coordinating ability of Cl^−^ and Br^−^ *versus* I^−^ that likely leads to the mineralization of **2** *versus* **3**.

If reactions similar to those above are performed with nickel or cobalt precursors (reaction of Ni(OAc)_2_·4H_2_O or Co(OAc)_2_·4H_2_O with H_3_BTC in [C_2_mim][Br] in a microwave-assisted or simple thermal reaction), the isostructural products [C_2_mim]_2_[Ni_3_(BTC)_2_(OAc)_2_] and [C_2_mim]_2_[Co_3_(BTC)_2_(OAc)_2_] are obtained (Lin *et al.*, 2006 ▸). In these compounds, the acetate ligands have not been completely displaced by the BTC^3−^ linkers. There are Ni_3_(OAc)_2_(*R*COO)_6_ SBUs (where *R*COO represents one carboxylate group of a BTC^3−^ linker) that have six outgoing linkages. Combined with the three linkages of BTC^3−^, a (3,6)-connected network is formed, with [C_2_mim]^+^ cations filling the cavities. The authors note that analogous reactions in [C_4_mim][Br] do not yield these products, and the [C_2_mim]^+^ cation is thus serving as a structure-directing agent.

The reaction of Co(OAc)_2_·4H_2_O with H_3_BTC in a mixture of [C_3_mim][Br] and [C_4_mim][NTf_2_] yields [C_3_mim]_2_[Co_2_(BTC)_2_(H_2_O)_2_] (Wang *et al.*, 2011 ▸). This compound contains Co_2_ units with two carboxylate groups bridging the Co atoms and two monodentate carboxylate groups and one water on each Co atom such that the Co_2_ unit forms a 6-connected node. These link to form an anionic [Co_2_(BTC)_2_(H_2_O)_2_]^2−^ framework, with [C_3_mim]^+^ cations within the galleries. This product can be contrasted with the [C_2_mim]_2_[Co_3_(BTC)_2_(OAc)_2_] obtained from a reaction in [C_2_mim][Br], as discussed above (Lin *et al.*, 2006 ▸).

### 3.3. Terephthalate, or benzene-1,4-dicarboxylate (BDC^2−^), MOFs   

In two fairly simple examples, the reactions of Ga_2_O_3_ and H_2_BDC (and other reactants) in [C_2_mim][Br] and [C_3_mim][Br] yielded [C_2_mim][Ga(BDC)_2_] and [C_3_mim][Ga(BDC)_2_], respectively (Li & Liu, 2015 ▸). The two compounds are iso­structural, with two-dimensional (2D) square frameworks of [Ga(BDC)_2_]^−^; each carboxylate group is coordinated in a mono­dentate manner to a tetrahedral Ga center. The [C_2_mim]^+^ and [C_3_mim]^+^ cations are in the open galleries, with slightly larger unit-cell parameters for the [C_3_mim]^+^ com­pound.

**Figure 4:**
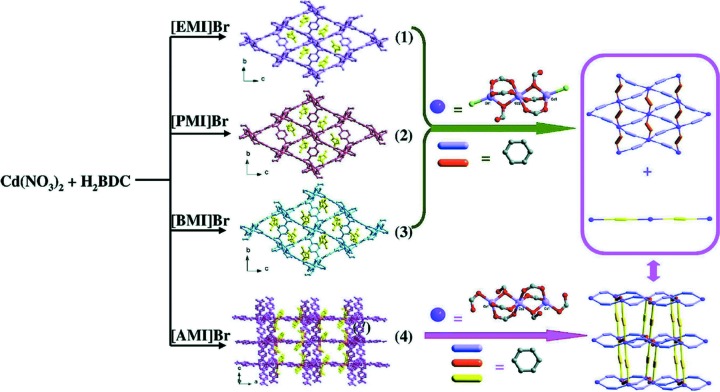
Structures of [C_*n*_mim]_2_[Co_3_(BDC)_3_Br_2_] (*n* = 2, 3, and 4 for EMI, PMI, and BMI, respectively) and [C_5_mim]_2_[Co_3_(BDC)_4_]. From Xu *et al.* (2014 ▸). Published by The Royal Society of Chemistry. Used with permission.

The products of the reaction of Co(NO_3_)_2_·6H_2_O with H_2_BDC in a series of imidazolium ILs, [C_*n*_mim][Br] (*n* = 2, 3, 4, 5), shows an interesting dependence on the size of the cation (Xu *et al.*, 2014 ▸). The three ILs with smaller cations all yield isostructural [C_*n*_mim]_2_[Co_3_(BDC)_3_Br_2_], while [C_5_mim][Br] yields [C_5_mim]_2_[Co_3_(BDC)_4_]. The [C_*n*_mim]_2_[Co_3_(BDC)_3_Br_2_] compounds contain Co_3_(*R*COO)_6_Br_2_ SBUs consisting of three Co atoms in a linear arrangement, with three carboxylate groups forming the bridges between pairs of Co atoms and the two end Co atoms capped by bromide ligands (see Fig. 4 ▸). The six BDC^2−^ linkers thus project outward roughly in one plane, leading to 2D nets of [Co_3_(BDC)_3_Br_2_]^2−^, as shown in Fig. 4 ▸. Because the [Co_3_(BDC)_3_Br_2_]^2−^ framework is 2D, it can accommodate a range of cation sizes in the cavities, from [C_2_mim]^+^ to [C_4_mim]^+^, with some changes in the in-plane structural parameters and expansion in the third dimension. However, at the largest cation size examined, [C_5_mim]^+^, a different MOF is obtained, [C_5_mim]_2_[Co_3_(BDC)_4_], which has a three-dimensional framework. It contains Co_3_ units very similar to the other compounds, with six BDC^2−^ linkers similarly directed outward to form a 2D framework. In place of the terminal bromide ligands are now two BDC^2−^ carboxylate groups, and those serve as pillars to connect the 2D layers into a 3D framework, as shown in Fig. 4 ▸. It is not obvious what causes the change in favored structure from 2D [C_4_mim]_2_[Co_3_(BDC)_3_Br_2_] to 3D [C_5_mim]_2_[Co_3_(BDC)_4_], but the change does show the strong structure-directing effect of the IL, since the addition of one methylene group to the cation results in a significant structural change. When similar reactions were performed in ILs with chloride or iodide anions, all the products were 2D MOFs [C_*n*_mim]_2_[Co_3_(BDC)_3_ *X*_2_] (*n* = 2, 3, 4, 5; *X* = Cl, I) isostructural with compounds **1**–**3** in Fig. 4 ▸ (Zhang *et al.*, 2016*b* ▸).

Another example of the reaction of Co(NO_3_)_2_·6H_2_O with H_2_BDC in [C_2_mim][Br], in this case with the addition of imidazole, shows the influence that additional additives can have on the MOF formed (Wang *et al.*, 2011 ▸). The product is Co_3_(BDC)_3_(imidazole)_2_, which contains Co_3_ units with a total of six bridging carboxylate groups, as in the MOFs of Fig. 4 ▸, but with the ends of the Co_3_ units capped by imidazole rather than bromide ligands, resulting in a neutrally charged framework. In addition, these 6-connected nodes are joined by the BDC^2−^ linkers to form a 3D network, in contrast to the 2D anionic framework of [C_2_mim]_2_[Co_3_(BDC)_3_Br_2_] in Fig. 4 ▸.

The reaction of Cd(NO_3_)_2_·4H_2_O with H_2_BDC in [C_4_mim][Br] yields [C_4_mim]_2_[Cd_3_(BDC)_3_Br_2_], which is iso­structural with the analogous cobalt compound described above (Liao & Huang, 2006 ▸). Similar reactions of Zn(NO_3_)_2_·6H_2_O with H_2_BDC in [C_2_mim][Cl] and [C_4_mim][Cl] yield [C_2_mim]_2_[Zn_3_(BDC)_3_Cl_2_] and [C_4_mim]_2_[Zn_3_(BDC)_3_Cl_2_], respectively (Tapala *et al.*, 2014 ▸). Both compounds contain 2D anionic [Zn_3_(BDC)_3_Cl_2_]^2−^ sheets constructed from Zn_3_ units with six radiating BDC linkers, as in compounds **1**–**3** of Fig. 4 ▸. The [Zn_3_(BDC)_3_Cl_2_]^2−^ sheets of the two MOFs are ‘isoreticular’ (3,6) 2D networks of the **hxl** type, but while [C_2_mim]_2_[Zn_3_(BDC)_3_Cl_2_] crystalizes in the monoclinic *P*2_1_/*n* space group like the analogous Co and Cd compounds, [C_4_mim]_2_[Zn_3_(BDC)_3_Cl_2_] crystalizes in the triclinic space group *P* 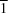 due to a different packing of the [Zn_3_(BDC)_3_Cl_2_]^2−^ layers. These results were replicated and extended to ionothermal syntheses in [C_*n*_mim][*X*] (*n* = 2, 3, 4, 5; *X* = Cl, Br, I) (Zhang *et al.*, 2015 ▸). The analogous 2D frameworks [C_*n*_mim]_2_[Zn_3_(BDC)_3_ *X*_2_] are formed for all cases *except* [C_4_mim][Cl], [C_5_mim][Cl], and [C_5_mim][Br]. For those three cases, the product was a 3D framework with the simple formula Zn(BDC)(H_2_O). In Zn(BDC)(H_2_O), each Zn atom is five-coordinated by four monodentate carboxyl­ate groups and one coordinated water molecule, such that the Zn centers function as 4-connected nodes. The BDC linkers bind to four separate Zn atoms through their four O atoms, so that they too act as 4-connected linkers. The overall topology is of the **pts** type (O’Keeffe *et al.*, 2008 ▸).

Reactions of LnCl_3_·6H_2_O (Ln = Sm, Eu, Tb) with H_2_BDC in [C_2_mim][Br] at 443 K for 4 d yields MOFs of two different structures, namely [C_2_mim]_2_[Sm_2_(BDC)_3_(H_2_BDC)Cl_2_] and [C_2_mim][Ln_2_(μ_2_-Cl)(BDC)_3_] (Ln = Eu, Tb) (Cao *et al.*, 2014 ▸). In both cases, there are chloride anions coordinated to the lanthanide (Ln) atom (originating from the LnCl_3_·6H_2_O reactant), rather than the bromide ions that are much more abundant from the IL, presumably due to the fact that the hard Lewis-acid Ln^3+^ ion favors the harder Cl^−^ base over the softer Br^−^ base. In the [Sm_2_(BDC)_3_(H_2_BDC)Cl_2_]^2−^ framework, there are Sm_2_(*R*COO)_6_(*R*COOH)Cl_2_ nodes, with all of the carboxylate groups bidentate and several bridging, such that each Sm center is nine-coordinated. The eight connections from the Sm_2_ SBU do not form the commonly observed **bcu** net topology, but instead form a new type of 8-connected net (Cao *et al.*, 2014 ▸). For the Eu and Tb MOFs, there are infinite chains of Ln_2_(μ_2_-Cl)(*R*COO)_6_ SBUs, with carboxylate groups bridging to form the chains and with six BDC^2−^ linkers (per Ln_2_ unit) extending in two dimensions, forming an overall **hex** network.

Blue crystals of the MOF [C_2_C_2_im][NaCu(BDC)_2_] (C_2_C_2_im = 1,3-diethylimidazolium) were isolated by the reaction of Cu(NO_3_)_2_·3H_2_O and H_2_BDC at 453 K in the mixed IL solvent system of [C_2_mim][BF_4_] and [C_2_mim][l-lactate] (Xiahou *et al.*, 2013 ▸). This raises the obvious question of the origin of the C_2_C_2_im^+^ and Na^+^ cations. According to the authors of the study, Na^+^ is present as an impurity in the [C_2_mim][BF_4_] IL. The C_2_C_2_im is formed *in situ* during the ionothermal synthesis by dealkylation and re-alkylation of [C_2_mim]^+^ cations. The structure of [C_2_C_2_im][NaCu(BDC)_2_] consists of infinite chains of alternating Na^+^ and Cu^2+^ ions bridged by carboxylate groups, with the BDC^2−^ linkers bridging to other Na^+^–Cu^2+^ chains to form a 3D framework of **pcu** topology. The formation of [C_2_C_2_im][NaCu(BDC)_2_] rather than [C_2_mim][NaCu(BDC)_2_] or an alternative [C_2_mim]^+^-containing MOF shows again the strong structure-directing effect of the IL cation, as the thermodynamic drive to form [C_2_C_2_im][NaCu(BDC)_2_] apparently overwhelms the fact that [C_2_mim]^+^ is present in much higher concentration, especially at the beginning of the reaction.

### 3.4. Other linking groups   

Three compounds of composition [C_2_mim]_2_[*M*_3_(*iso*-BDC)_4_] (*M* = Mn, Co, Ni; *iso*-BDC = benzene-1,3-dicarboxylate) were prepared ionothermally in [C_2_mim][Br] solvent (Chen *et al.*, 2011 ▸). All three structures contain *M*_3_(OOC*R*)_8_ SBUs that serve as 8-connected nodes. The Co and Ni compounds are isostructural, with a **bcu** network topology. In the Mn compound, the *M*_3_(OOC*R*)_8_ SBUs have a different arrangement, but again form a network with **bcu** topology.

The lanthanum-containing MOF [C_2_mim][La(5-NO_2_-*iso*-BDC)BrCl] was obtained from the reaction of LaCl_3_·*n*H_2_O with 5-NO_2_-*iso*-H_2_BDC in [C_2_mim][Br] (Chen *et al.*, 2009 ▸), while the bimetallic lanthanum–cobalt MOF [C_2_mim]_2_[La_2_Co(5-CH_3_-*iso*-BDC)_2_(OAc)_2_(CH_3_SO_3_)_4_] was obtained from LaCl_3_·*n*H_2_O and Co(OAc)_2_·4H_2_O with 5-CH_3_-*iso*-H_2_BDC in [C_2_mim][CH_3_SO_3_] (Chen *et al.*, 2009 ▸).

The MOF [C_3_′mim]_2_[Mg_3_(1,4-NDC)_4_(1-mim)_2_(H_2_O)_2_]·2H_2_O ([C_3_′mim]^+^ = 1-allyl-3-methylimidazolium and 1,4-NDC = naph­thalene-1,4-dicarboxylate) was obtained from the ionothermal reaction of Mg(NO_3_)_2_·6H_2_O and H_2_-1,4-NDC in [C_3_′mim][Cl] at 433 K for 6 d (Wu *et al.*, 2011 ▸). The nodes comprise Mg_3_(*R*COO)_8_(1-mim)_2_(H_2_O)_2_ SBUs, with three Mg ions in a linear arrangement bridged by carboxylate groups and water molecules and a 1-mim ligand on each of the terminal Mg ions. The nodes are linked through eight 1,4-NDC units, forming a **bcu** network topology. [C_3_′mim][Cl] is known to thermally decompose to allyl chloride and 1-mim (Hao *et al.*, 2010 ▸), and that is the presumed source of 1-mim in the MOF.

The ionothermal reaction of Zn(NO_3_)_2_·6H_2_O and benzene-1,2,4,5-tetracarboxylic acid (H_4_BTetC) in [C_2_mim][Br] at 433 K for 7 d gave colorless crystals of [C_2_mim]_2_[Zn_3_(BTetC)_2_]·2H_2_O (Ji *et al.*, 2011 ▸). The MOF contains both left- and right-handed helices of Zn–BTetC chains, crosslinked by BTetC^4−^ to form a 3D framework, with [C_2_mim]^+^ cations in the galleries. The MOF is a ferroelectric with a ‘colossal’ dielectric constant.

The colorless MOF [C_4_dmim][Mg_3_(OBA)_3_(HOBA)] [[C_4_dmim]^+^ = 1-butyl-2,3-dimethylimidazolium and H_2_OBA = 4,4′-oxybis(benzoic acid)] was synthesized by the reaction of Mg(NO_3_)_2_·6H_2_O and H_2_OBA at 433 K for 6 d in [C_4_dmim][Br] (Wu *et al.*, 2012 ▸). The structure consists of linear chains of Mg^2+^ bridged by carboxylate groups, with the OBA^2−^ and HOBA^−^ linkers acting as struts to connect the chains into a 3D framework.

The purple homochiral MOF [C_2_mim][Co_2_(d-cam)_2_(OAc)] (H_2_ d-cam = d-camphoric acid) was synthesized by the reaction of Co(OAc)_2_·6H_2_O and H_2_ d-cam at 393 K for 7 d in [C_2_mim][Br] (Chen *et al.*, 2008 ▸). The MOF is ‘homochiral’ because only the d enantiomer of the chiral linker camphorate is present. The structure is comprised of Co_2_(*R*COO)_4_ paddlewheel SBUs, where the *R*COO carboxylate groups are one end of a camphorate ligand. The Co_2_ units are thus linked into a 2D square network. Bound to the apical site of each Co atom of the Co_2_ SBUs is a single O atom of an acetate ligand, while the other O atom of the acetate ligand is bound to a Co_2_ SBU in another layer, such that the acetate ions acts as pillars joining the 2D nets into a 3D anionic framework. As usual, the [C_2_mim]^+^ cations reside in the open regions in the framework. The same group reported several indium–camphorate MOFs, such as [C_2_mim][In(d-cam)_2_], in [C_2_mim][ethyl sulfate] (Zhang *et al.*, 2008 ▸).

## 4. Anion structure-directing effects   

In an ionothermal synthesis, it is more common for the cation of an IL, rather than the anion, to be incorporated into the MOF structure and act as a template and structure-directing agent. That could be viewed as a consequence of the fact that the metals (or metal clusters) that comprise the nodes of the MOF will usually accommodate a coordination number that is higher than their cationic charge (for example, Zn^2+^ with a coordination number of 4 in a tetrahedral complex). It is thus possible for the metal atom to bond to more negatively charged linkers than its positive charge, leading to a node (and a framework) with a net negative charge. That negative charge can be balanced by the cation of the IL if the MOF forms as an open framework that accommodates the cation (and the cation thus acts as a template). However, there are many cases in which the IL anion becomes part of the MOF, usually when the anion is somewhat coordinating (such as a halide) and binds to the metal center in the MOF. Examples of anion incorporation, and other forms of anion structure-directing effects, are discussed in this section.

In one interesting study, the IL anion is not incorporated into any of the MOFs synthesized, yet the identity of the IL anion controls the MOF that is formed (Lin *et al.*, 2007*b* ▸). The reaction of Co(OAc)_2_·4H_2_O with H_3_BTC was carried out in three different ILs or IL combinations, *viz.* neat [C_2_mim][Br], a 1:1 mixture of [C_2_mim][Br] and [C_2_mim][NTf_2_], and neat [C_2_mim][NTf_2_], leading to three different MOFs, namely [C_2_mim]_2_[Co_3_(BTC)_2_(OAc)_2_], [C_2_mim][Co(BTC)], and [Co_5_(OH)_2_(OAc)_8_]·2H_2_O, respectively. When the reaction was performed in [C_2_mim][NTf_2_] with added 2,2′-bipyridine (2,2′-bipy), a fourth MOF was formed, [C_2_mim][Co_2_(H_2_BTC)_3_(HBTC)(2,2′-bipy)_2_]. [C_2_mim]_2_[Co_3_(BTC)_2_(OAc)_2_] is iso­structural with the previously reported nickel analog (Lin *et al.*, 2006 ▸) (as discussed above), while [C_2_mim][Co(BTC)] is isostructural with [C_2_mim][Cd(BTC)] (Liao *et al.*, 2006 ▸) and [C_2_mim][Mn(BTC)] (Xu *et al.*, 2013 ▸), both also discussed above. [Co_5_(OH)_2_(OAc)_8_]·2(H_2_O) has an extended framework structure with most of the bridges between Co atoms formed by acetate ligands and could be considered a MOF for that reason, even though it incorporates none of the intended BTC^3−^ linking ligand. In fact, [Co_5_(OH)_2_(OAc)_8_]·2H_2_O had been synthesized previously by the hydrolytic decomposition of Co(acac)_3_ (acac = acetyl­acetonate) in THF/H_2_O at 528 K (Kuhlman *et al.*, 1999 ▸). In [C_2_mim][Co_2_(H_2_BTC)_3_(HBTC)(2,2′-bipy)_2_], the 2,2′-bipy ligands leave four coordination sites available on each Co atom, which are filled by monodentate carboxyl­ate groups from the BTC ligand, forming 2D (4,4)-nets, joined by hydrogen bonds.

A set of cadmium MOFs were ionothermally synthesized in reactions in which reaction/decomposition of both the IL BF_4_^−^ anion and an added ligand, 4-picoline (4-pic or 4-methylpyridine), created components of the MOF (Xie *et al.*, 2012 ▸). The reaction of Cd(NO_3_)_2_·4H_2_O with 4-pic in [C_4_mim][BF_4_] yielded [Cd_3_F(ina)_4_(4-pic)_3_][BF_4_] (ina = iso­nicotinate or pyridine-4-carboxylate). The source of the fluoride anion is hydrolysis of the BF_4_^−^ anion, while oxidation of 4-pic furnishes the isonicotinic acid, with nitrate as the most likely oxidizing agent. Both fluoride and isonicotinate were incorporated into the MOF framework. The structure of [Cd_3_F(ina)_4_(4-pic)_3_][BF_4_] contains [Cd_3_F(*R*COO)_3_(pyr)_3_]^2+^ SBUs, where *R*COO represents the carboxyl­ate end of isonicotinate and pyr represents the pyridyl end. These SBUs have three Cd^2+^ ions in a triangle centered around the fluoride anion, with the three carboxyl­ate groups bridging the edges of the triangle. Each Cd^2+^ ion is capped by one pyridyl group of an isonicotinate ligand, so there are six linkers projecting hexagonally from each SBU to form a hexagonal 2D framework of [Cd_3_F(ina)_3_]^2+^. [These SBUs bear some similarity to the Cr_3_O(*R*COO)_6_(H_2_O)_3_ SBUs of well-known MOF MIL-101 (Férey *et al.*, 2005 ▸).] The carboxyl­ate group of a fourth ina ligand bridges two Cd^2+^ ions and forms a link to another 2D layer *via* its pyridyl functionality, thus forming an overall 3D MOF. The net positive charge per formula unit is balanced by BF_4_^−^ anions in the interstitial regions, and 4-pic ligands fill out the octahedral coordination sphere of the Cd cations. Two other MOFs with similar 2D frameworks, *i.e.* [Cd_3_F(ina)_3_(4,4′-bipy)_2_(4-pic)_2_][BF_4_]_2_·4,4′-bipy·2H_2_O and [Cd_3_F(ina)_3_(4,4′-bipy)_3_][BF_4_]_2_·4,4′-bipy·2H_2_O, were made with added 4,4′-bipyridine (4,4′-bipy), such that 4,4′-bipy bridges between the layers.

Two related MOFs, [C_2_mim]_2_[Co(H_2_O)_2_(O_2_CCF_2_CF_2_CO_2_)_2_] (O_2_CCF_2_CF_2_CO_2_ = tetrafluorosuccinate) and [C_2_mim]_2_[Co_3_(H_2_O)_4_(O_2_CCF_2_CF_2_CF_2_CO_2_)_4_] (O_2_CCF_2_CF_2_CF_2_CO_2_ = hexafluoroglutarate), could be synthesized only in a mixture of [C_2_mim][Br] and [C_2_mim][NTf_2_], and not in either pure IL (Hulvey *et al.*, 2009 ▸). The polar and coordinating bromide anion serves to solubilize the metal salt, while the NTf_2_^−^ anion decreases the polarity of the solvent to match the fluorinated linkers. In [C_2_mim]_2_[Co(H_2_O)_2_(O_2_CCF_2_CF_2_CO_2_)_2_], the Co centers are octahedrally coordinated by four carboxylate groups and two *trans* water molecules, with the bridging tetra­fluoro­succinate ligands serving to form a 2D anion sheet with [C_2_mim]^+^ cations in the cavities. [C_2_mim]_2_[Co_3_(H_2_O)_4_(O_2_CCF_2_CF_2_CF_2_CO_2_)_4_] contains linear Co_3_ units in Co_3_(H_2_O)_4_(*R*CO_2_)_8_ SBUs. Two of the water molecules bridge Co atoms, while the other two cap the terminal Co atoms. The Co_3_ SBUs are then linked into 2D sheets by the hexafluoroglutarate linkers.

In MOFs in which the organic linking group is neutral (and which would therefore not be MOFs under Yaghi’s definition), it is inevitable that the anions of either the IL or the metal salt starting material will be incorporated into the final MOF, and thus have some structure-directing effect. Two early examples are of this type. The reaction of Cu(NO_3_)_2_·3H_2_O and 1,3-bis(4-pyridyl)propane (bpp) in [C_4_mim][BF_4_] yields the framework compound [Cu(bpp)][BF_4_] (Jin *et al.*, 2002 ▸). The framework has a +1 charge per formula unit; note that the Cu^2+^ ions have been reduced to Cu^+^ during the ionothermal reaction, most likely by the bpp ligand. In [Cu(bpp)][BF_4_], the Cu^+^ ions are effectively two-coordinated, with a linear coordination of the two pyridyl groups, forming one-dimensional polymers of Cu(bpp)^+^. There are short Cu^+^–Cu^+^ contacts at 3.002(2) Å and, if those are considered, there is a 2D framework with BF_4_^−^ anions interstitial between the layers. A similar synthesis involves the reaction of Cu(NO_3_)_2_·3H_2_O and 2,4,6-tris(4-pyridyl)-1,3,5-triazine (tpt) in [C_4_mim][BF_4_] to yield [Cu_3_(tpt)_4_][BF_4_]_3_·
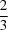
tpt·5H_2_O (Dybtsev *et al.*, 2004 ▸). Once again the copper has been reduced from Cu^2+^ to Cu^+^. All of the Cu^I^ ions are tetrahedrally coordinated by the tpt ligands, which function as trigonal planar linkages. The overall net is of **ctn-a** topology.

The reactions of CuCl_2_ with tetrazole-1-acetic acid (1-Htza) in [C_4_mim][Br], [C_4_mim][BF_4_], and [C_4_mim][NTf_2_] yielded several different coordination networks (Chen *et al.*, 2014 ▸). Most relevant are the 2D framework [Cu_2_(1-tza)_4_]Br·H_3_O·
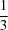
H_2_O and the 3D framework [Cu_2_(1-tza)_4_][BF_4_]·H_3_O·H_2_O.

## 5. Cation and anion incorporation/combined control   

There are several examples of ionothermal syntheses of MOFs in which both the cation and anion of the IL are incorporated into the MOF, or otherwise have some structure-directing effect. The most common way that both IL ions become part of the MOF is when the anion coordinates to the metal center of the resulting anionic MOF framework, while the IL cation acts as a template for the open galleries for the MOF, where it acts as a counter-ion to balance the charge of the framework. Examples of this type and others are discussed below.

A set of six Zn–BTC MOFs synthesized in [C_*n*_mim][Br] ILs demonstrate the structure-directing effects of the IL cation and also the anion (in comparison with another synthesis in [C_4_mim][I]), and also the importance of the reaction stoichiometry (Xu *et al.*, 2007 ▸). The reactions all used the precursors Zn(NO_3_)_2_·6H_2_O and H_3_BTC in various [C_*n*_mim][Br] (*n* = 2–5) at 433 K for 5 d. Reaction in [C_2_mim][Br] yielded two polymorphs of [Zn_3_(BTC)_2_(H_2_O)_2_]·2H_2_O, with the structure obtained depending upon the starting stoichiometric ratio of Zn(NO_3_)_2_·6H_2_O to H_3_BTC (3:1 and 4:1). Both polymorphs of [Zn_3_(BTC)_2_(H_2_O)_2_]·2H_2_O are 3D frameworks, but their structures will not be described in detail here. A third reaction in [C_2_mim][Br], with a 6:1 molar ratio of Zn(NO_3_)_2_·6H_2_O to H_3_BTC, yields a MOF with the simple stoichiometry [C_2_mim][Zn(BTC)]. [C_2_mim][Zn(BTC)] has a 2D anionic framework of Zn(BTC)^−^, with [C_2_mim]^+^ cations in the open channels. It should be noted that yet another synthesis in [C_2_mim][Br] with a 5:1 ratio of Zn(NO_3_)_2_·6H_2_O to H_3_BTC, discussed above in §2, yields the 3D MOF Zn_4_(BTC)_2_(μ_4_-O)(H_2_O)_2_ (Xu *et al.*, 2008*c* ▸). Synthesis in [C_3_mim][Br] yields [C_3_mim][Zn(BTC)], which has a 3D anionic framework, demonstrating the strong effect of a small change in the steric bulk of the IL cation. Synthesis in [C_4_mim][Br] yields [C_4_mim]_2_[Zn_4_(BTC)_3_(OH)(H_2_O)_3_] and synthesis in [C_5_mim][Br] yields [C_5_mim][Zn_2_(BTC)(OH)Br], both of which contain anionic 3D frameworks. The latter compound is distinguished from the others in this series by having bromide bound to zinc as part of the framework. Finally, a separate publication describes a similar ionothermal synthesis in the iodide IL [C_4_mim][I], which yields the 3D MOF [C_4_mim][Zn_2_(BTC)(OH)I] (Xu *et al.*, 2008*b* ▸). The presence of iodide leads to the creation of a novel SBU, namely the tetrazinc node Zn_4_(OH)_2_I_2_(*R*CO_2_)_6_.

Moving one position downward in the periodic table from Zn to Cd, three Cd–BTC MOFs were prepared ionothermally in [C_*n*_mim][*X*] (*n* = 2, 3; *X* = Cl, Br, I) (Xu *et al.*, 2008*a* ▸). The products included [C_2_mim][Cd_2_(BTC)Cl_2_] (from [C_2_mim][Cl]), [C_2_mim][Cd(BTC)] (from [C_2_mim][Br] or [C_2_mim][I]), and [C_3_mim][Cd(BTC)] (from [C_3_mim][*X*]). [C_2_mim][Cd(BTC)] had been reported previously (Liao *et al.*, 2006 ▸), as discussed in §3.2. When the more strongly coordinating chloride-containing IL is used, [C_2_mim][Cd_2_(BTC)Cl_2_] is obtained. It contains Cd_2_Cl_4_ units linked by BTC^3−^ to form a 3D framework. If the IL cation is changed to [C_3_mim]^+^, the MOF [C_3_mim][Cd(BTC)] is obtained, which has a [Cd(BTC)]^−^ framework with the same topology as that in [C_2_mim][Cd(BTC)], though with a small change in bonding (one carboxylate group changes its coordination from bidentate to monodentate). Interestingly, in no case is a halide incorporated into the anionic framework, showing that a small change in the steric bulk of the IL cation determines whether the IL anion becomes part of the MOF.

**Figure 5:**
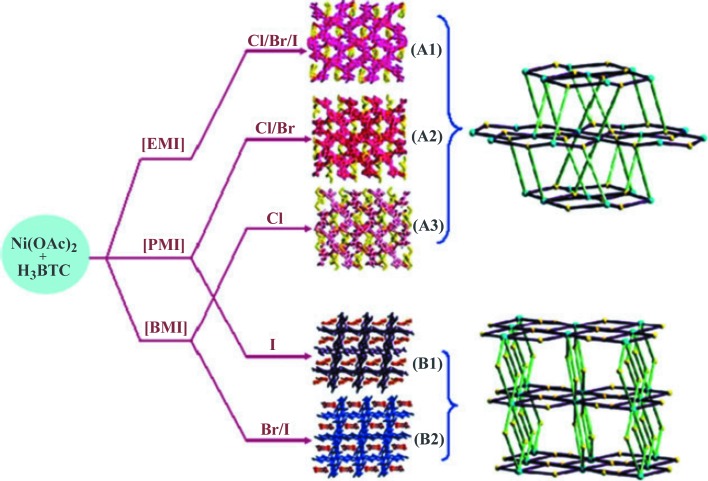
Reaction scheme, structures, and schematic representation of the topologies of [C_*n*_mim]_2_[Ni_3_(BTC)_2_(OAc)_2_] [*n* = 2 (**A1**), 3 (**A2**), or 4 (**A3**)] and [C_*n*_mim]_2_[Ni_3_(H-BTC)_4_(H_2_O)_2_] [*n* = 2 (**B1**) or 4 (**B2**)]. [EMI] = [C_2_mim]^+^, [PMI] = [C_3_mim]^+^, and [BMI] = [C_4_mim]^+^. In the structure drawings, imidazolium cations are shown in yellow (**A1**, **A2**, and **A3**) and orange (**B1** and **B2**). In the topology drawings, the cyan and yellow spheres represent the Ni_3_ units and BTC^2−^ linkers, respectively. The 2D layers are shown in purple. From Xu *et al.* (2009 ▸). Published by The Royal Society of Chemistry. Used with permission.

The reaction of Ni(OAc)_2_·4H_2_O with H_3_BTC in the nine different ILs [C_*n*_mim][*X*] (*n* = 2, 3, 4; *X* = Cl, Br, I) gave five different MOFs of two different structure types, as shown schematically in Fig. 5 ▸ (Xu *et al.*, 2009 ▸). The products are [C_*n*_mim]_2_[Ni_3_(BTC)_2_(OAc)_2_] [*n* = 2 (**A1**), 3 (**A2**), or 4 (**A3**)] and [C_*n*_mim]_2_[Ni_3_(H-BTC)_4_(H_2_O)_2_] [*n* = 3 (**B1**) or 4 (**B2**)]. In no case is any halide incorporated into the MOF, but for the [C_3_mim]^+^ and [C_4_mim]^+^ ILs, the identity of the halide determines which structure type is obtained: [C_3_mim][Cl] and [C_3_mim][Br] give structure type **A**, while [C_3_mim][I] gives structure type **B**; [C_4_mim][Cl] gives structure type **A**, while [C_4_mim][Br] and [C_4_mim][I] give structure type **B** (all shown in Fig. 5 ▸). In both structure types, there are linear Ni_3_ units bridged by BTC^3−^ carboxylate groups, which are then linked to form 2D layers. Those 2D layers are in turn linked to form 3D frameworks, as shown schematically in Fig. 5 ▸. The main point of interest in this set of MOFs is the formation of two dif­ferent MOF structure types, in some cases with the same cation. The cavities within the anionic framework of the **B** structure are larger than those in the **A** structure, so it is un­surprising that the **B** structure tends to form for the larger cations. However, the formation of two different structures con­taining the same cation (**A2** and **B1**; **A3** and **B2**) indicates that there may be a kinetic effect where the IL halide anion induces the crystallization of one structure type in favor of the other. Another possibility is that the relative basicity of the halide anions of the IL exerts thermodynamic control on the structure type obtained: structure **B**, in which the BTC is singly pro­ton­ated, is favored when less basic anions are used.

## 6. Other structure-directing effects   

In one set of Co–HBTC MOFs, it is the hydrogen bonding of various added amines that directs the structure that is formed (Lin *et al.*, 2008 ▸). All of the synthetic reactions involved Co(OAc)_2_·4H_2_O and H_3_BTC in [C_2_mim][Br] with an added amine, including 4,4′-bipy, imidazole, and 1,4-diazabicyclo­octane (DABCO). The products were [C_2_mim][Br][Co_2_(HBTC)_2_(4,4′-bipy)_3_], [C_2_mim][Br][Co(HBTC)(4,4′-bipy)_2_]·4,4′-bipy, [C_2_mim][Co(BTC)(imidazole)], and [C_2_mim]_2_[Co(BTC)_2_(H_2_DABCO)]. All of the compounds have 2D Co–BTC coordination networks, but have different 3D structures directed by both the coordination of the amines to the Co centers (for the 4,4′-bipy and imidazole compounds), and hydrogen bonding between the amines and residual carboxylic acid functionalities (or carboxylate group, in the case of imidazole).

Another MOF shows the templating effects of hydrated potassium cations. The reaction of Cd(NO_3_)_2_·4H_2_O, H_4_BTetC, and KCl in [C_2_mim][Br] yielded [K_2_(H_2_O)_8_][Cd_3_(BTetC)_2_] (Ji *et al.*, 2008 ▸). The porous anionic framework of [Cd_3_(BTetC)_2_]^2−^ contains Cd_3_ units bound by eight carboxylate groups of four BTetC^4−^ linkers. These Cd_3_ units are linked in three dimensions to leave open one-dimensional (1D) channels, which contain [K_2_(H_2_O)_8_]^2+^ chains. Each K^+^ is eight-coordinate, with two monodentate waters of coordination, four bridging waters to form the chains, and two carboxylate groups that are also part of the [Cd_3_(BTetC)_2_]^2−^ network.

There are two reported ionothermal syntheses of a single enantiomer of chiral MOFs using ILs containing chiral and enantiomerically pure anions. In neither case is the IL anion incorporated as part of the MOF, but the chirality of the anion directs the MOF structure toward only one enantiomer. In the first example, Ni(OAc)_2_·4H_2_O was reacted with H_3_BTC in [C_4_mim][l-aspartate] to form the homochiral MOF [C_4_mim]_2_[Ni(HBTC)_2_(H_2_O)_2_], which crystallizes in the space group *P*4_1_2_1_2 (Lin *et al.*, 2007*a* ▸). The nodes of the structure consist of Ni coordinated by four monodentate carboxylate groups and two water molecules, and these 4-connected nodes are linked to form a diamondoid (**dia**) network. The chirality of the MOF results from the 4_1_ screw axis, which leads to a helical arrangement of the Ni SBUs. If the reaction is performed in [C_4_mim][d-aspartate], the same compound is obtained with the opposite chirality. In the second example of this type of chiral induction, the reaction of Cu(NO_3_)_2_·3H_2_O with H_2_-1,4-NDC in a mixture of [C_2_mim][l-lactate] and [C_2_mim][BF_4_] yielded [C_2_mim][NaCu(1,4-NDC)_2_] (Liu *et al.*, 2013 ▸). The Na^+^ originates from an impurity in the [C_2_mim][BF_4_] IL. The compound crystallizes in the space group *P*4_1_, and it is once again the 4_1_ screw axis that is the origin of the chirality.

## 7. Conclusions   

A large number of MOF and MOF-like framework materials have been synthesized in IL solvents. The ILs very often exert structure-directing effects, commonly through the incorporation of the IL cation (as a charge-balancing ion within the galleries of the MOF), anion (usually coordinated to a metal center within an overall negatively charged MOF framework), or both. Even when no part of the IL is incorporated into the MOF, the IL can exert a structure-directing effect, as occurs when different IL anions direct the crystallization of different MOF polymorphs, or when a chiral IL anion directs the formation of one enantiomer of a chiral MOF.

In the ionothermal syntheses described herein, the ILs very often contain halide anions (most commonly chloride and bromide, but also iodide), certainly much more than is common in other applications of ILs, where weakly coordinating and chemically inert anions, such as PF_6_^−^ and NTf_2_^−^, have found more widespread use. The moderate coordinating ability of the halides makes them useful in the solubilization of the metal-containing precursors for MOFs and crystallization of the MOFs themselves. The coordinating ability of the halides can also lead to their incorporation into the MOF framework. The cations of the ILs in ionothermal MOF syntheses are most commonly of the [C_*n*_mim]^+^ type, which is likely due to their easy availability and the fact that their asymmetric structure leads to melting points below 373 K for most [C_*n*_mim]^+^ halides. This points to new opportunities for non-imidazolium ILs in MOF synthesis, where different cations would impose new steric constraints on the MOFs and likely lead to new materials. The room-temperature ionic liquid trihexyltetradecylphosphonium chloride, for example, contains a cation that is significantly larger and of a different shape from [C_*n*_mim]^+^ cations, and it and related ILs are good candidates for future syntheses of new MOFs by ionothermal methods.

## Figures and Tables

**Table 1:** Metal–organic frameworks and the ionic liquids in which they were synthesized

Metal–organic framework	Ionic liquid	Reference
		
§2: neither IL cation nor anion incorporation		
Zn_4_(BTC)_2_(μ_4_-O)(H_2_O)_2_	[C_2_mim][Br]	Xu *et al.* (2008*c* ▸)
[NBu_4_]_2_[Cu(BBTZ)_2_(Mo_8_O_26_)]	[C_2_mim][Br]	Fu *et al.* (2012 ▸, 2011 ▸)
(1-mim)Ni(BDC)	[C_3_mim][Br]	Hogben *et al.* (2006 ▸)
Zn_3_(BDC)_3_(1-mim)_2_	[C_4_mim][BF_4_]	Wang *et al.* (2008 ▸)
Co[4,5-di(*N*-acetyl)­amino-2-methylimdiazo­late]_2_	[NEt_4_][2-methyl-4,5-di­cyano­imidazolate]	Mondal *et al.* (2014 ▸)
Zn(C_2_O_4_)(C_3_N_2_H_4_)	[C_2_mim][NTf_2_]	Martins *et al.* (2010 ▸)
Zn(C_3_N_2_H_3_)_2_	[C_2_mim][NTf_2_]	Martins *et al.* (2010 ▸)
Zn(OAc)(C_3_N_2_H_3_)	[C_2_mim][NTf_2_]	Martins *et al.* (2010 ▸)
Zn_4_(C_3_N_2_H_3_)_8_(C_3_N_2_H_4_)	[C_2_mim][NTf_2_]	Martins *et al.* (2010 ▸)
*M*_3_[Fe(CN)_6_]_2_ (*M* = Ni, Cu, Co)	[C_*n*_mim][BF_4_], [C_*n*_mim][Cl] (*n* = 2, 4, 10)	Clavel *et al.* (2006 ▸); Larionova *et al.* (2008 ▸)
		
§3: IL cation incorporation/templating		
[C_2_mim][Cd(BTC)]	[C_2_mim][Br]	Liao *et al.* (2006 ▸)
[C_*n*_mim][Mn(BTC)] (*n* = 2 or 3)	[C_2_mim][*X*] or [C_3_mim][*X*] (*X* = Cl, Br, I)	Xu *et al.* (2013 ▸)
[C_2_mim]_2_[*M*_3_(BTC)_2_(OAc)_2_] (*M* = Ni, Co)	[C_2_mim][Br]	Lin *et al.* (2006 ▸)
[C_3_mim]_2_[Co_2_(BTC)_2_(H_2_O)_2_]	[C_3_mim][Br]–[C_4_mim][NTf_2_] mixture	Wang *et al.* (2011 ▸)
[C_*n*_mim][Ga(BDC)_2_] (*n* = 2 or 3)	[C_*n*_mim][Br] (*n* = 2 or 3)	Li & Liu (2015 ▸)
[C_*n*_mim]_2_[Co_3_(BDC)_3_Br_2_] (*n* = 2, 3, 4)	[C_*n*_mim][Br] (*n* = 2, 3, 4)	Xu *et al.* (2014 ▸)
[C_5_mim]_2_[Co_3_(BDC)_4_]	[C_5_mim][Br]	Xu *et al.* (2014 ▸)
[C_*n*_mim]_2_[Co_3_(BDC)_3_ *X*_2_] (*n* = 2, 3, 4, 5; *X* = Cl, I)	[C_*n*_mim][*X*] (*n* = 2, 3, 4, 5; *X* = Cl, I)	Zhang *et al.* (2016*b* ▸)
Co_3_(BDC)_3_(imidazole)_2_	[C_2_mim][Br], imidazole	Wang *et al.* (2011 ▸)
[C_4_mim]_2_[Cd_3_(BDC)_3_Br_2_]	[C_4_mim][Br]	Liao & Huang (2006 ▸)
[C_2_mim]_2_[Zn_3_(BDC)_3_Cl_2_]	[C_2_mim][Cl]	Tapala *et al.* (2014 ▸)
[C_4_mim]_2_[Zn_3_(BDC)_3_Cl_2_]	[C_4_mim][Cl]	Tapala *et al.* (2014 ▸)
[C_*n*_mim]_2_[Zn_3_(BDC)_3_ *X*_2_]	[C_n_mim][*X*] (*n* = 2, 3, 4, 5; *X* = Cl, Br, I; except for the 3 ILs listed below	Zhang *et al.* (2015 ▸)
Zn(BDC)(H_2_O)	[C_4_mim][Cl], [C_5_mim][Cl], and [C_5_mim][Br]	Zhang *et al.* (2015 ▸)
[C_2_mim]_2_[Sm_2_(BDC)_3_(H_2_BDC)Cl_2_]	[C_2_mim][Br]	Cao *et al.* (2014 ▸)
[C_2_mim][Ln_2_(μ_2_-Cl)(BDC)_3_] (Ln = Eu and Tb)	[C_2_mim][Br]	Cao *et al.* (2014 ▸)
[C_2_C_2_im][NaCu(BDC)_2_]	[C_2_mim][BF_4_]–[C_2_mim][L-lactate] mixture	Xiahou *et al.* (2013 ▸)
[C_2_mim]_2_[*M*_3_(*iso*-BDC)_4_] (*M* = Mn, Co, Ni)	[C_2_mim][Br]	Chen *et al.* (2011 ▸)
[C_2_mim][La(5-NO_2_-*iso*-BDC)BrCl]	[C_2_mim][Br]	Chen *et al.* (2009 ▸)
[C_2_mim]_2_[La_2_Co(5-CH_3_-*iso*-BDC)_2_(OAc)_2_(CH_3_SO_3_)_4_]	[C_2_mim][CH_3_SO_3_]	Chen *et al.* (2009 ▸)
[C_3_′mim]_2_[Mg_3_(1,4-NDC)_4_(1-mim)_2_(H_2_O)_2_]·2H_2_O	[C_3_′mim][Cl]	Wu *et al.* (2011 ▸)
[C_2_mim]_2_[Zn_3_(BTetC)_2_]·2H_2_O	[C_2_mim][Br]	Ji *et al.* (2011 ▸)
[C_4_dmim][Mg_3_(OBA)_3_(HOBA)]	[C_4_dmim][Br]	Wu *et al.* (2012 ▸)
[C_2_mim][Co_2_(D-cam)_2_(OAc)]	[C_2_mim][Br]	Chen *et al.* (2008 ▸)
[C_2_mim][In(D-cam)_2_]	[C_2_mim][ethyl sulfate]	Zhang *et al.* (2008 ▸)
		
§4: IL anion incorporation or structure-directing effects		
[C_2_mim]_2_[Co_3_(BTC)_2_(OAc)_2_]	[C_2_mim][Br]	Lin *et al.* (2007*b* ▸)
[C_2_mim][Co(BTC)]	[C_2_mim][Br]–[C_2_mim][NTf_2_] mixture	Lin *et al.* (2007*b* ▸)
[Co_5_(OH)_2_(OAc)_8_]·2H_2_O	[C_2_mim][NTf_2_]	Lin *et al.* (2007*b* ▸)
[C_2_mim][Co_2_(H_2_BTC)_3_(HBTC)(2,2′-bipy)_2_]	[C_2_mim][NTf_2_], 2,2′-bi­pyridine	Lin *et al.* (2007*b* ▸)
[Cd_3_F(ina)_4_(4-pic)_3_][BF_4_]	[C_4_mim][BF_4_], 4-picoline	Xie *et al.* (2012 ▸)
[Cd_3_F(ina)_3_(4,4′-bipy)_2_(4-pic)_2_][BF_4_]_2_·4,4′-bipy·2H_2_O	[C_4_mim][BF_4_], 2,2′-bi­pyridine	Xie *et al.* (2012 ▸)
[Cd_3_F(ina)_3_(4,4′-bipy)_3_][BF_4_]_2_·4,4′-bipy·2H_2_O	[C_4_mim][BF_4_], 2,2′-bi­pyridine	Xie *et al.* (2012 ▸)
[C_2_mim]_2_[Co(H_2_O)_2_(O_2_CCF_2_CF_2_CO_2_)_2_]	[C_2_mim][Br]–[C_2_mim][NTf_2_] mixture	Hulvey *et al.* (2009 ▸)
[C_2_mim]_2_[Co_3_(H_2_O)_4_(O_2_CCF_2_CF_2_CF_2_CO_2_)_4_]	[C_2_mim][Br]–[C_2_mim][NTf_2_] mixture	Hulvey *et al.* (2009 ▸)
[Cu(bpp)][BF_4_]	[C_4_mim][BF_4_]	Jin *et al.* (2002 ▸)
[Cu_3_(tpt)_4_][BF_4_]_3_· 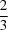 tpt·5H_2_O	[C_4_mim][BF_4_]	Dybtsev *et al.* (2004 ▸)
[Cu_2_(1-tza)_4_]Br·H_3_O· 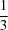 H_2_O	[C_4_mim][Br]	Chen *et al.* (2014 ▸)
[Cu_2_(1-tza)_4_][BF_4_]·H_3_O·H_2_O	[C_4_mim][BF_4_]	Chen *et al.* (2014 ▸)
		
§5: IL cation and anion incorporation or combined structural control		
[Zn_3_(BTC)_2_(H_2_O)_2_]·2H_2_O	[C_2_mim][Br]	Xu *et al.* (2007 ▸)
[C_2_mim][Zn(BTC)]	[C_2_mim][Br]	Xu *et al.* (2007 ▸)
[C_3_mim][Zn(BTC)]	[C_3_mim][Br]	Xu *et al.* (2007 ▸)
[C_4_mim]_2_[Zn_4_(BTC)_3_(OH)(H_2_O)_3_]	[C_4_mim][Br]	Xu *et al.* (2007 ▸)
[C_5_mim][Zn_2_(BTC)(OH)Br]	[C_5_mim][Br]	Xu *et al.* (2007 ▸)
[C_4_mim][Zn_2_(BTC)(OH)I]	[C_4_mim][I]	Xu *et al.* (2008*b* ▸)
[C_2_mim][Cd_2_(BTC)Cl_2_]	[C_2_mim][Cl]	Xu *et al.* (2008*a* ▸)
[C_2_mim][Cd(BTC)]	[C_2_mim][Br] or [C_2_mim][I]	Xu *et al.* (2008*a* ▸)
[C_3_mim][Cd(BTC)]	[C_3_mim][*X*] (*X* = Cl, Br, or I)	Xu *et al.* (2008*a* ▸)
[C_*n*_mim]_2_[Ni_3_(BTC)_2_(OAc)_2_] (*n* = 2, 3, 4)	[C_2_mim][*X*] (*X* = Cl, Br, I); [C_3_mim][*X*] (*X* = Cl, Br); [C_4_mim][Cl]	Xu *et al.* (2009 ▸)
[C_*n*_mim]_2_[Ni_3_(HBTC)_4_(H_2_O)_2_] (*n* = 3, 4)	[C_3_mim][I]; [C_4_mim][*X*] (*X* = Br, I)	Xu *et al.* (2009 ▸)
		
§6: other structure-directing effects		
[C_2_mim][Br][Co_2_(HBTC)_2_(4,4′-bipy)_3_]	[C_2_mim][Br], 4,4′-bi­pyridine	Lin *et al.* (2008 ▸)
[C_2_mim][Br][Co(HBTC)(4,4′-bipy)_2_]·4,4′-bipy	[C_2_mim][Br], 4,4′-bi­pyridine	Lin *et al.* (2008 ▸)
[C_2_mim][Co(BTC)(imidazole)]	[C_2_mim][Br], imidazole	Lin *et al.* (2008 ▸)
[C_2_mim]_2_[Co(BTC)_2_(H_2_DABCO)]	[C_2_mim][Br], DABCO	Lin *et al.* (2008 ▸)
[K_2_(H_2_O)_8_][Cd_3_(BTetC)_2_]	[C_2_mim][Br], KCl	Ji *et al.* (2008 ▸)
[C_4_mim]_2_[Ni(HBTC)_2_(H_2_O)_2_]	[C_4_mim][L-aspartate]	Lin *et al.* (2007*a* ▸)
[C_4_mim]_2_[Ni(HBTC)_2_(H_2_O)_2_] (opposite chirality)	[C_4_mim][D-aspartate]	Lin *et al.* (2007*a* ▸)
[C_2_mim][NaCu(1,4-NDC)_2_]	[C_2_mim][L-lactate]–[C_2_mim][BF_4_] mixture	Liu *et al.* (2013 ▸)
